# A Cross-Sectional Survey of Cannabis Use and Utility Among Patients Experiencing Dizziness

**DOI:** 10.3390/brainsci16040360

**Published:** 2026-03-27

**Authors:** Pardise Elmi, Dorsa Mavedatnia, Gabriel Berberi, Marc Lawrence, Angelina Tohmé, Xinyuan Hong, Daniel Lelli, Darren Tse

**Affiliations:** 1 Department of Otolaryngology-Head and Neck Surgery, University of British Colombia, Vancouver, BC V6T 1Z4, Canada; 2 Department of Otolaryngology-Head and Neck Surgery, University of Toronto, Toronto, ON M5S 1A1, Canada; 3 Faculty of Medicine and Health Sciences, McGill University, Montreal, QC H3A 2B3, Canada; 4 Division of Neurology, Department of Internal Medicine, University of Ottawa, Ottawa, ON K1H 8M5, Canada; 5 Ottawa Hospital Research Institute, Ottawa, ON K1H 8L6, Canada; 6 Department of Otolaryngology-Head and Neck Surgery, Queen’s University, Kingston, ON K7L 3N6, Canada; 7 Department of Otolaryngology-Head and Neck Surgery, University of Ottawa, Ottawa, ON K1H 8L6, Canada

**Keywords:** dizziness, cannabis, vestibular disorders

## Abstract

**Highlights:**

**What are the main findings?**

- Patients with dizziness demonstrate interest in using cannabis as a therapeutic option.
- Cannabis may offer benefits for dizziness-related symptoms that significantly impact quality of life in patients.

**What are the implications of the main findings?**

- Although there is interest in cannabis-based treatments for dizziness, patients report learning about it through non-medical sources, demonstrating limited clinical guidance and the need for clinician-led counselling and standardized patient education.

**Abstract:**

**Background/Objective**: Dizziness is managed using various therapies, including lifestyle changes, nutritional supplementation, pharmaceutical therapies, and physical therapy, each offering differing efficacy. With legalization of cannabis in Canada, patients are exploring its use in treating their dizziness and related symptoms. Limited knowledge exists regarding usage patterns, forms, doses, and effects on these symptoms. The current study aims to examine cannabis use in patients experiencing dizziness. **Methods**: We conducted a cross-sectional study at the Ottawa Hospital outpatient neuro-otology clinic. Eligible participants included new patients presenting with a primary complaint of dizziness and follow-up patients reporting new-onset dizziness. Participants completed a questionnaire capturing demographic data, dizziness and related symptoms, attitudes toward cannabis use, consumption patterns, and its perceived effects on their symptoms. **Results**: Of 154 participants, 118 (77%) expressed willingness to consider cannabis for dizziness and 78 (51%) reported previous cannabis use. Of those patients, 44 (56%) consumed it recreationally, largely via smoking (29/78, 37%). Approximately 21% of these patients reported a moderate–large subjective relief from dizziness after use of cannabis. The most common diagnoses amongst cannabis users were migraine/vestibular migraine (24%), persistent postural perceptive dizziness (22%), and benign paroxysmal positional vertigo (17%). Other related symptoms relieved by cannabis included sleep (28/78, 36%), emotional difficulties (17/78, 22%), neck pain/stiffness (14/78, 18%) and headaches/migraines (9/78, 12%). **Conclusions**: There is generally a positive attitude towards cannabis use in treating dizziness amongst patients, with a subset of patients reporting a subjective improvement of dizziness and its related symptoms, such as sleep and emotional difficulties.

## 1. Introduction

Dizziness is a common complaint amongst patients of all ages, with a prevalence of 35.6% [1]. Characterized by unsteadiness, light headedness, or a false sense of motion, affecting balance and spatial awareness, its etiologies include vestibular and non-vestibular causes. Vestibular causes include peripheral vestibular disorders (e.g., BPPV) and central disorders (e.g., migraines). Non-vestibular causes include cardiovascular and psychiatric conditions. Dizziness limits independence and daily functioning, significantly impairing quality of life [2,3,4,5,6,7]. Among patients experiencing dizziness of vestibular origin, 19% avoided leaving their home, 41% took sick leave, and 40% could not perform daily activities [8]. Annual dizziness care costs an estimated $31 million CAD in Ontario, Canada, and $48 billion USD in the United States [9,10]. Understanding and addressing dizziness is crucial for improving quality of life and reducing healthcare burden.

Managing dizziness is complex, requiring varied approaches. While vestibular suppressants (e.g., anti-emetics, antihistamines, benzodiazepines) are used to treat dizziness and its related symptoms like nausea, vomiting, and anxiety, they often cause adverse effects [11,12]. These effects are exacerbated in older adults, including extrapyramidal symptoms, sedation, urinary retention, tachyarrhythmia, and respiratory depression [13]. These medications may also slow vestibular compensation and prolong symptoms [12]. This leaves a gap in practice for effective pharmacologic management of dizziness symptoms while minimizing adverse reactions. Cannabinoids are increasingly used to regulate nausea, vomiting, and anxiety [14,15,16]. However, evidence on cannabinoid use for dizziness remains limited.

Cannabinoids bind to receptors in the central nervous system and peripheral tissues [17]. The endocannabinoid system regulates pain, memory, movement, metabolism, and immunity [17]. Tetrahydrocannabinol (THC) and cannabidiol (CBD) are the primary clinically relevant cannabinoids. CBD offers neuroprotection, anti-inflammation, and anti-oxidation without psychoactive effects, though its mechanism is unclear [18]. The endocannabinoid system comprises two primary G protein-coupled receptor subtypes including type-1 cannabinoid (CB1) and type-2 cannabinoid (CB2) [19]. CB1 receptors are more abundant and widely expressed in the central nervous system, functioning to regulate synaptic transmission and inhibit neurotransmitter release [19,20,21]. CB2 receptors were largely thought to be peripheral immune receptors, mostly expressed on immune cells [19,21]. However, the recent literature has shown that lower levels of CB2, in comparison to CB1, are also expressed in the brain across various regions [22,23,24]. CB2 receptors primarily play a role in immune modulation, neuroinflammation, and neuroprotection [23,24,25]. Endocannabinoids are synthesized during hyper-excitable states and epileptiform activity, aiding seizure control in treatment-resistant epilepsy [26]. Cannabis has also demonstrated effect in reducing the occurrence of dizziness, nausea, vomiting, anxiety, insomnia, and irritability [27]. In pediatric epilepsy patients, CBD use through oil-based extracts was linked to a reduction in dizziness, going from 35.9% to 0% [27]. Its effect on dizziness is likely due to its role on CB1 receptors in the vestibular nuclear complex [26,27,28,29,30].

With its central regulation and anti-inflammatory, anti-emetic, and anxiolytic properties, cannabis is a promising treatment option for patients with dizziness [14,15,16,26]. Cannabis has a more favourable side effect profile than vestibular suppressants [18]. Although CBD may cause fatigue, diarrhea, and appetite changes, it is a safe adjunct therapy for Parkinson’s disease, Crohn’s disease, sleep disorders, anxiety, and chemotherapy side effects [28]. In otolaryngology, cannabis has been studied to manage tinnitus, blepharospasm, obstructive sleep apnea, auditory and sinonasal symptoms, head and neck radiation side effects, and cancer-related psychological impacts [31,32,33,34]. It is generally well tolerated, even with chronic use and high dosages [29]. Since Canada’s legalization of cannabis in 2018, its medical use and public perception have evolved. The objective of this study is to assess patient attitudes and patterns of cannabis use among patients with dizziness at a Canadian tertiary care hospital. We hypothesize that patients view cannabis positively and report symptom relief from its use.

## 2. Materials and Methods

### 2.1. Study Design and Ethics Approval

This prospective cross-sectional study was conducted at the Ottawa Hospital’s outpatient neuro-otology clinic. The study was approved by the Ottawa Hospital Science Network Research Ethics Board (OHSN-REB: 20230034-01H). The date of ethics approval was 16 February 2023.

### 2.2. Study Population

New patients presenting with a primary complaint of dizziness to the specialized neuro-otology and vestibular clinics of the Ottawa Vestibular Program at the Ottawa Hospital were eligible for inclusion. This includes patients referred for diagnostic opinions or management from primary care, emergency departments across eastern Ontario, and other specialists. Follow-up patients with new complaints of dizziness, and no prior involvement in this study, were also included. Patients were approached and recruited to participate in the study during their visit at the clinic. All patients provided written consent before participating.

### 2.3. Questionnaire Design

Patients completed a questionnaire about their symptoms, previous history of cannabis use, and attitudes towards cannabis use prior to their appointment. Questions were in a multiple choice, multiple response, Likert scale, and open-ended format, and were completed in the waiting room prior to their appointment. Although their physicians did not have access to their answers, after the consultation, they entered a diagnosis and comorbid factors to complete all the necessary information for the study. The primary outcomes of the questionnaire were patient attitudes towards cannabis, the patterns of cannabis use amongst patients, and its effects on self-reported dizziness symptoms.

### 2.4. Statistical Analysis

Questionnaires were anonymized prior to data entry into a spreadsheet. Patients were divided into 3 subgroups depending on their diagnosis of a central/functional vestibular disorder (CFVD), peripheral vestibular disorder (PVD), or non-vestibular disorder (NVD). Descriptive statistics including mean, median, range, frequencies, and percentages were calculated through Microsoft Excel (Microsoft Corp., Redmond, WA, USA). Figures were generated using RStudio (version 4.3.3, R Foundation for Statistical Computing, Vienna, Austria). Open-ended survey responses were reviewed, and pertinent quotes were selected and added to the results. The results were separated into three categories based on the survey topics. These sections include attitudes towards cannabis, cannabis use patterns, and subjective assessment of cannabis effects on dizziness symptoms.

## 3. Results

A total of 272 patients were eligible and approached to participate in the study. The response rate was 57%, with 154 patients completing the questionnaire.

The mean age of participants was 51 years, with 111 females (72%), 40 males (26%), and two individuals (1%) who did not identify as either (Table 1). The average duration of dizziness was 52 months, with 32% of patients experiencing daily episodes (49/154). A large portion of respondents had a primary diagnosis of vestibular migraines (48/154, 31%), persistent postural–perceptual dizziness (PPPD) (29/154, 19%) or benign paroxysmal positional vertigo (BPPV) (25/154, 16%). The most common comorbid condition in patients was migraine (22/154, 14%), excluding patients with a primary diagnosis of vestibular migraines. Demographic data was comparable between peripheral vestibular disorder (PVD) and central/functional vestibular disorder (CFVD). The sample size in the non-vestibular disorder (NVD) subgroup was small, with few responses in each answer category. All the results are summarized in Table 1.

### 3.1. Attitudes Towards Cannabis

Approximately 77% (118/154) of participants reported that they would consider cannabis-related medications to treat their dizziness (Table 2) with 51% of the total population reporting having previously tried cannabis (78/154). Among respondents who had not previously used cannabis, 67% (45/67) indicated that they would consider it. Among those with prior cannabis use, 92% (72/78) expressed willingness to consider cannabis-based treatments, and 8% (6/78) stated that they would not. Nearly half the patients (69/154, 45%) stated that they would consider cannabis-related medications because they had been informed of its possible symptom-relief potential, either through others or external sources. Almost a third of patients (*n* = 46, 30%) indicated that they would consider it because other therapies have not provided adequate relief.

Overall, patients were curious about the literature surrounding cannabis-related medications and open to the possibility of new treatments. While some patients felt open to trying cannabis-related medications based on suggestions from their peers, others expressed that they would be most motivated to try if recommended by their doctor.

Regarding concerns surrounding cannabis-related medications, 70 (46%) patients indicated fear of psychosocial side effects, 47 (31%) expressed concerns for physical health side effects, and 42 (27%) were apprehensive due to the cost. Thirty-six (24%) indicated “other” with some patients sharing a general disinterest in cannabis use. Others were more fearful about functional impairment and physical side effects including inability to drive, weight gain, interactions with existing medications, and worsening dizziness symptoms. Patients also shared fear of the social stigma associated with cannabis and how their peers might react.

Patients reported learning about cannabis through personal research, professional and occupational exposure, or through their social circle. Some participants shared that they have learned through university pharmacology courses, podcasts, and documentaries. Others worked in fields that exposed them to cannabis, such as physiotherapy and the federal civil service.

Overall, reported attitudes towards cannabis were comparable between PVD and CFVD subgroups. The NVD subgroup had a small sample size, with few responses in each answer category. All the results are summarized in Table 2.

### 3.2. Cannabis Use Patterns Amongst Patients with Dizziness

Of the patients having previously used cannabis, 57 (78%) reported using it prior to the onset of their dizziness (Table 3). Most participants used cannabis for recreation (44/78, 56%), sleep (30/78, 39%), chronic pain (18/78, 23%), emotional difficulties (16/78, 21%), and dizziness/vertigo/imbalance (9/78, 12%). Eight patients selected “other” and reported using cannabis for post-concussive symptoms, musculoskeletal pain management, and nausea/vomiting relief.

Participants recorded using cannabis through a variety of routes (Table 4). Notably, 29 people using it through smoking (37%), 28 through infused foods or drinks (36%), 22 through infused oil (28%), nine through vaporizers (12%), and three through applied creams (4%). Of those who smoked cannabis, the average smoked daily quantity was 0.26 g with a mode of 0.13 g (12/29, 41%).

For most questions, reported patterns of cannabis use and relation to dizziness were comparable between the PVD and CFVD subgroups. The NVD subgroup had a small sample size, with few responses in each answer category. The results are summarized in Table 3.

### 3.3. Subjective Assessment of Cannabis Effects on Dizziness Symptoms

**Figure 1:**
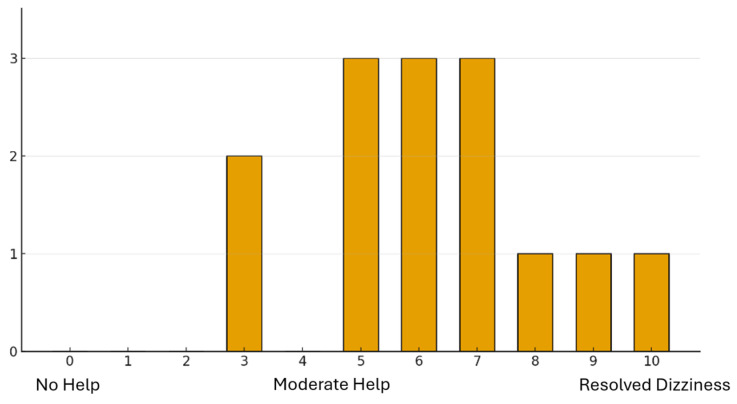
Subjective effect of cannabis use on patients’ reported dizziness symptoms. This bar graph illustrates the distribution of self-reported dizziness symptom relief among patients with previous cannabis use. The *x*-axis represents the perceived effectiveness of cannabis on a scale from 0 (no help) to 10 (resolved dizziness). The *y*-axis indicates the number of patients reporting each level of effect.

Of the 78 individuals who have tried cannabis, 14 (18%) indicated that it had improved their dizziness while 46 (59%) indicated that it had not. Of those with improved symptoms, 64% (9/14) rated their improvement between 5 and 7/10 (Figure 1) and 21% (3/14) between 8 and 10/10, indicating that they felt their cannabis use led to dizziness symptom resolution.

Overall, 28 (36%) reported improved sleep, 17 (22%) reduced emotional difficulties, 14 (18%) noted improvements in neck pain and stiffness, and 11 (14%) reported improvements in dizziness, vertigo and imbalance. However, sixteen respondents (21%) indicated that cannabis use improved none of the dizziness-related symptoms listed on the questionnaire. Written comments reported that higher percentages of THC were found to worsen dizziness and/or cause symptoms such as paranoia and anxiety while lower THC or CBD percentages were found to be helpful.

Reported cannabis dosage, methods of use and effects on dizziness were comparable between the PVD and CFVD subgroups. The NVD subgroup had a small sample size, with few responses in each answer category. The results are summarized in Table 4.

## 4. Discussion

This is the first study to investigate the use of cannabis amongst patients with dizziness. This study suggests a general willingness in patients with dizziness from both central (CFVD) and peripheral vestibular pathologies (PVD) to utilize and learn more about cannabis therapy as a treatment option. Understanding patient attitudes toward cannabis is essential for researching its use in treating dizziness.

The endocannabinoid system (ECS) is essential for regulating pain, emotion, memory, neural protection, and reducing central hyperexcitability and inflammation [17,18,26,27]. This in part is controlled through the inhibition of endocannabinoid uptake, regulating CB1 receptors, and activating serotonergic receptors. In the central nervous system, endocannabinoids and cannabinoids bind to CB1 receptors, inhibiting the release of excitatory neurotransmitters and reducing neuronal hyperexcitability [26]. The effect of CBD on dizziness is partially thought to be due to the existence of CB1 and CB2 receptors in the vestibular nuclei [26]. The effect of a highly potent CB1 receptor agonist (CP-55) has demonstrated strong inhibition of medial vestibular nuclei neurons in guinea pigs, suggesting that CB1 receptors are located presynaptically and modulate neuronal excitability by reducing glutamate release [26]. Additionally, CB2 receptors have been seen in the medial vestibular and cochlear nuclei of rats [35,36]. The confirmation of functioning CB1 receptors in the vestibular nuclei along with the presence of CB2 receptors further substantiates the crucial role of the ECS in vestibular reflexes.

While effective in managing various medical conditions, a common concern with medicinal cannabis is the inconsistency of its regulation. Many products contain higher THC levels than federally permitted and less CBD than labelled, complicating clinical research due to strain variability and inconsistent THC:CBD ratios [37]. These discrepancies may contribute to variable side effect profiles, such as dizziness reported in some clinical trials [38,39,40,41]. It is theorized that dizziness from cannabis is due to its vasodilatory effects, resulting in postural hypotension [42]. However, this effect is shown to be transient, diminishing after 1–2 days of repeat exposure with tolerance. Nonetheless, our questionnaire did not capture this possibility in our patients consuming cannabis. Chronic users show a decrease in heart rate and disappearance of orthostatic hypotension [42,43]. Cannabinoid Hyperemesis Syndrome is an emerging gastrointestinal condition associated with frequent and long-term use of cannabis [44]. Its estimated prevalence of 3.43 per 100,000 based on emergency visits in Ontario, Canada, remains far lower than that of dizziness at 35.6% [1,45]. Accordingly, patients using cannabis to relieve symptoms of dizziness should be educated on the potential risks but not discouraged from its use without further individualized assessment and consideration of alternative treatments for their symptoms.

Variations in THC:CBD ratios and formulations may also play a role in inconsistent reports of dizziness with cannabis use. Whole plant cannabis with 12.5% THC was not associated with dizziness while sublingual sprays with similar THC:CBD ratios were [46,47]. In patients with migraine, THC:CBD combinations have been reported to provide greater pain relief and symptom freedom than other formulations. Given the overlap between migraine and vestibular migraine, these findings raise the possibility that similar cannabis formulations may also influence dizziness-related symptoms in patients with vestibular migraine [48]. This is further suggested by the fact that patients in the current study have interestingly reported noticeable differences in the effect of cannabis on their dizziness, owing to different formulations and THC:CBD contents. In order to accurately understand the role of cannabis in dizziness, standardization of cannabis formulation and content is imperative along with follow-ups to determine how its side effect profiles can change over time.

There is a well-established, likely reciprocal link between dizziness, depression and anxiety [8,14,15,49,50,51]. In our study, 52% of cannabis users reported doing so to manage emotional difficulties such as anxiety and stress [52]. Physical neurotologic conditions were found to induce psychiatric pathologies just as frequently as primary psychopathologies caused dizziness [52]. Neuro-otologic conditions were also found to worsen pre-existing anxiety and depression [52]. Though the pathophysiology linking vestibular symptoms and psychological distress is unclear, overlapping neuroanatomical regions and neurotransmitters seen in both the vestibular system and in emotional regulation are thought to be the cause [50]. Cannabis has been shown to reduce anxiety, depression, and pain, improve sleep and increase overall self-reported quality of life in those diagnosed with clinical anxiety and depression, and this was corroborated in the present study [53]. Given the bi-directional relationship between vestibular symptoms and emotional distress, the perceived benefits we observed may reflect modulation of concomitant mood or sleep symptoms rather than a direct vestibular effect.

Tinnitus and dizziness are common complaints that often pose a challenge for physicians to treat effectively [31]. The pathophysiology of both symptoms is thought to be related to neuronal hyperexcitability. In a similar study as the present one, 96% of patients with tinnitus (*n* = 45) demonstrated a willingness to try cannabis-related medications, with 80% of cannabis users finding an improvement of their tinnitus and 37.5% an improvement in dizziness symptoms [31].

This study has some limitations. Despite using a large and diverse sample, recruiting all patients from the same tertiary clinics with 72% being females may limit the generalizability of our findings. Given the referral pattern to the Ottawa Vestibular Program, there is likely a bias towards patients with more chronic and recalcitrant dizziness. While our results appeared comparable between the PVD and CFVD subgroups, the sample size in NVD was too small to make conclusions regarding the generalizability of our findings to non-vestibular dizziness populations. The etiology, duration of symptoms, and possibility of confounding comorbidities among our population is quite variable, which impacts treatment response, patient perceptions and desperation for treatment. Additionally, the social stigma surrounding cannabis use may have also deterred the participation of patients with reservations about cannabis, introducing a potential response bias skewing results toward a pro-cannabis stance with contributions of recall bias from the self-reported nature of the data [54]. Lastly, our study only evaluated the positive effect of cannabis on dizziness, without considering its negative effects. Because our study assesses attitudes and self-reported experiences without a control group, standardized products, or diagnostic stratification, our findings provide descriptive evidence that motivates future studies. Diagnosis-specific trials controlling for confounders such as the co-occurrence of mood, anxiety and sleep disorders with standardized formulations and safety follow-up are needed to understand the direct or adjunctive role of cannabinoids in managing dizziness.

## 5. Conclusions

In conclusion, this cross-sectional study provides valuable new insight into cannabis use and attitudes among patients with dizziness. Though cannabis has not been studied in dizziness, its central action and its therapeutic properties, as well as its relationship with the vestibular system, show promise as a potential treatment option for patients with dizziness that should be further researched [17,18,26,27,28,29,30]. The results of our study demonstrate that patients are open to cannabis-based treatments, with some reporting decreased dizziness and relief of dizziness-related symptoms such as better sleep and improved mood. These findings should be interpreted as exploratory and hypothesis-generating rather than evidence of therapeutic efficacy. Randomized control trials are needed to establish optimal doses and forms of cannabis in treating dizziness to determine the long-term effects of cannabis use on dizziness and delineate the impacts of cannabis on various etiologies of dizziness.

## Figures and Tables

**Table 1:** Patient demographics and general dizziness information.

Demographic Categories	Total N = 154 (%)	PVD N = 35 (%)	CFVD N = 100 (%)	NVD N = 12 (%)	No Response
**Age**					N = 1
Mean, years	51.04	56.57	49.25	47.33	
Range, years	(16–80)	(26–80)	(16–78)	(28–69)	
Median, years	52	60	51	28	
**Sex**					N = 1
Female	111 (72.1)	24 (68.57)	78 (78.00)	7 (58.33)	
Male	40 (25.97)	11 (31.43)	19 (19.00)	5 (41.67)	
Other	2 (1.30)	-	2 (2.00)	-	
Prefer not to state	-	-	-	-	
**Duration of Dizziness Experienced**					N = 8
Mean, months	52	57.68	52.70	51.25	
Range, months	(1–550)	(1–550)	(1–432)	(1–180)	
Mode, months	24	24	24	12	
**Frequency of Dizziness**					N = 5
Less than once a month	19 (12.34)	8 (22.86)	8 (8.00)	1 (8.33)	
1–10 times a month	36 (23.38)	7 (20.00)	27 (27.00)	1 (8.33)	
More than 3 times a week	20 (12.99)	2 (5.71)	16 (16.00)	1 (8.33)	
Once a day	8 (5.19)	1 (2.86)	6 (6.00)	1 (8.33)	
Multiple times every day	49 (31.82)	16 (45.71)	24 (24.00)	6 (50.00)	
Constantly all the time	17 (11.04)	1 (2.86)	15 (15.00)	1 (8.33)	
Therapies Attempted *					N = 32
Vestibular physiotherapy	78 (50.65)	18 (51.43)	55 (55.00)	1 (8.33)	
Vestibular suppressants	71 (46.10)	15 (42.86)	51 (51.00)	2 (16.67)	
Antidepressants	36 (23.40)	4 (11.43)	32 (32.00)	-	
Steroids (e.g., prednisone)	13 (8.44)	5 (14.29)	6 (6.00)	-	
Acupuncture	22 (14.29)	6 (17.14)	13 (13.00)	1 (8.33)	
Massage/chiropractor	45 (29.22)	7 (20.00)	35 (35.00)	1 (8.33)	
Psychotherapy/counselling/cognitive behavioural therapy (CBT)	31 (20.13)	-	28 (28.28)	1 (8.33)	
**Main Diagnosis**					N = 27
Vestibular migraines	48 (31.17)	13 (37.14)	21 (21.00)	1 (8.33)	
Persistent postural–perceptual dizziness (3PD)	29 (18.83)	4 (11.43)	37 (37.00)	-	
Benign paroxysmal positional vertigo (BPPV)	25 (16.23)	2 (5.71)	31 (31.00)	-	
Not yet diagnosed	7 (4.55)	4 (11.43)	9 (9.00)	2 (16.67)	
Vestibular neuritis	3 (1.95)	-	4 (4.00)	2 (16.67)	
Bilateral vestibular hypofunction (BVH)	3 (1.95)	2 (5.71)	23 (23.00)	1 (8.33)	
Basilar migraine	3 (1.95)	2 (5.71)	13 (13.00)	2 (16.67)	
Postural orthostasis	2 (1.30)	8 (22.86)	38 (38.00)	4 (33.33)	
Meniere’s disease	2 (1.30)	7 (20.00)	33 (33.00)	3 (25.00)	
Syncope	2 (1.30)	4 (11.43)	18 (18.00)	1 (8.33)	
Anxiety related	1 (0.65)	13 (37.14)	21 (21.00)	1 (8.33)	
Multisensory gait dysfunction	1 (0.65)	4 (11.43)	37 (37.00)	-	
Autoimmune inner ear disease (AIED)	1 (0.65%)	2 (5.71)	31 (31.00)	-	
**Contributing comorbidities**					N = 113
Migraine	22 (14.28)	2 (5.71)	20 (20.00)	-	
Anxiety	5 (3.24)	-	4 (3.00)	1 (8.33)	
Vestibular migraines	5 (3.24)	-	5 (5.00)	-	
Other neurologic pathologies (i.e., BPPV migraines, SSNHL, BVH, cerebellar infarct)	4 (2.60)	2 (5.71)	2 (2.00)	-	
Chronic migraines	4 (2.60)	-	4 (4.00)	-	
Concussion	2 (1.30)	-	2 (2.00)	-	
Palpitations	1 (0.65)	-	-	1 (8.33)	
Chronic rhinosinusitis	1 (0.65)	-	1 (1.00)	-	

**Legend**. This table summarizes the patient demographics and the general information about their dizziness. * Some survey questions were multi-response, allowing patients to select more than one option. Patients had the choice not to answer some questions. PVD = peripheral vestibular disorder. CFVD = central/functional vestibular disorder. NVD = non-vestibular disorder.

**Table 2:** Patient attitudes towards cannabis.

Categories	Total N = 154 (%)	PVD N = 35 (%)	CFVD N = 100 (%)	NVD N = 12 (%)	No Response
**Would patients consider using cannabis-related medications to treat dizziness?**					N = 8
Yes	118 (76.62)	24 (68.57)	81 (81.00)	7 (58.33)	
No	28 (18.18)	7 (20.00)	15 (15.00)	5 (41.67)	
Concerns with trying cannabis-related medications: *					N = 20
Cost	42 (27.27)	9 (25.71)	28 (28.00)	3 (25.00)	
Physical health side effects (lung disease)	47 (30.52)	7 (20.00)	33 (33.00)	4 (33.33)	
Psychosocial side effects (psychosis, paranoia, etc.)	70 (45.45)	11 (31.43)	51 (51.00)	7 (58.33)	
Other	36 (23.38)	7 (20.00)	25 (25.00)	3 (25.00)	
What dizziness-related symptoms would patients consider cannabis-related treatments for? *					N = 5
Functional difficulties (concentration, brain fog)	87 (58.39)	19 (54.29)	57 (57.00)	6 (50.00)	
Emotional difficulties (depression, anxiety, fear)	77 (51.68)	19 (54.29)	50 (50.00)	4 (33.33)	
Headache	82 (55.03)	12 (34.29)	65 (65.00)	3 (25.00)	
Neck pain and/or stiffness	75 (50.34)	13 (37.14)	54 (54.00)	6 (50.00)	
Sleep	82 (55.03)	18 (51.43)	53 (53.00)	7 (58.33)	
I would not consider using cannabis for any of these symptoms	22 (14.77)	6 (17.14)	12 (12.00)	3 (25.00)	
I don’t have any of these symptoms	6 (4.03)	3 (8.57)	3 (3.00)	-	
Reasons patients would consider cannabis-related medication: *					N = 24
Have heard that cannabis-related medication may provide relief	69 (44.81)	12 (34.29)	49 (49.00)	5 (41.67)	
Other therapies have not provided adequate relief	46 (29.87)	8 (22.86)	31 (31.00)	4 (33.33)	
Other therapies have undesired or intolerable side effects	15 (9.74)	1 (2.86)	12 (12.00)	1 (8.33)	
Already taking cannabis-related medications for other reasons	8 (5.19)	3 (8.57)	4 (4.00)	-	
Resources used to learn about cannabis: *					N = 9
I don’t know anything about cannabis	38 (24.68)	11 (31.43)	23 (23.00)	1 (8.33)	
Doctor or nurse	21 (13.64)	4 (11.43)	16 (16.00)	1 (8.33)	
Friend/family member	63 (40.91)	11 (31.43)	46 (46.00)	5 (41.67)	
Nutritionist	-	-	-	-	
Naturopath/herbalist	3 (1.95)	-	2 (2.00)	-	
Social media (Facebook, Twitter, etc.)	28 (18.18)	3 (8.57)	19 (19.00)	4 (33.33)	
Medicinal cannabis store	9 (5.84)	1 (2.86)	6 (6.00)	-	
Recreational cannabis store	19 (12.34)	5 (14.29)	12 (12.00)	-	
Pamphlet/handout	8 (5.19)	-	7 (7.00)	1 (8.33)	
Newspaper/magazine article	37 (24.02)	7 (20.00)	28 (28.00)	2 (16.67)	
TV/radio advertisement	8 (5.19)	3 (8.57)	5 (5.00)	-	
Website/blogs	23 (14.94)	4 (11.43)	16 (16.00)	2 (16.67)	
**How many patients have previously used cannabis?**					N = 4
Have used cannabis	78 (51.00)	16 (45.71)	47 (47.00)	6 (50.00)	
Have not used cannabis	72 (46.75)	18 (51.43)	51 (51.00)	5 (41.67)	

**Legend**. This table summarizes patient attitudes towards cannabis. * Some survey questions were multi-response, allowing patients to select more than one option. Patients had the choice not to answer some questions. PVD = peripheral vestibular disorder. CFVD = central/functional vestibular disorder. NVD = non-vestibular disorder.

**Table 3:** Patterns of cannabis use and timing in relation to dizziness symptoms.

Categories	Total N = 78 (%)	PVD N = 18 (%)	CFVD N = 51 (%)	NVD N = 5 (%)	No Response
**Timing of cannabis use in relation to dizzy symptoms:**					N = 0
Cannabis used before the start of dizzy symptoms	57 (73.08)	14 (77.78)	34 (66.67)	5 (100.00)	
Cannabis used after the start of dizzy symptoms	21 (26.92)	4 (22.22)	16 (31.37)	1 (20.00)	
**Most recent use of cannabis:**					N = 0
Over a year ago	33 (43.31)	4 (22.22)	24 (47.06)	3 (60.00)	
9–12 months ago	5 (6.41)	1 (5.56)	3 (5.88)	1 (20.00)	
6–9 months ago	3 (3.85)	1 (5.56)	2 (3.92)	-	
3–6 months ago	4 (5.13)	1 (5.56)	3 (5.88)	-	
1–3 months ago	11 (14.1)	3 (16.67)	7 (13.73)	1 (20.00)	
Less than 1 month ago	5 (6.41)	2 (11.11)	3 (5.88)	-	
Last week	9 (11.54)	4 (22.22)	3 (5.88)	-	
This week	1 (1.28)	-	1 (1.96)	-	
Yesterday	4 (5.13)	1 (5.56)	2 (3.92)	1 (20.00)	
Today	3 (3.85)	1 (5.56)	2 (3.92)	-	
Currently high	-	-	-	-	
Reasons for using cannabis: *					N = 6
Recreational use	44 (56.41)	15 (83.33)	23 (45.10)	3 (60.00)	
Dizziness/vertigo/imbalance	9 (11.54)	1 (5.56)	8 (15.69)	-	
Functional difficulties (concentration, fatigue, brain fog)	5 (6.41)	1 (5.56)	2 (3.92)	1 (20.00)	
Emotional difficulties (depression, anxiety, fear)	16 (20.51)	2 (11.11)	12 (23.53)	1 (20.00)	
Headache or migraines	7 (8.97)	-	6 (11.76)	-	
Neck pain and/or stiffness	9 (11.54)	2 (11.11)	6 (11.76)	-	
Sleep	30 (38.46)	7 (38.89)	18 (35.29)	3 (60.00)	
Chronic pain	18 (23.08)	2 (11.11)	13 (25.49)	2 (40.00)	
Diagnosed medical condition	1 (1.28)	-	1 (1.96)	-	
**Frequency of cannabis use:**					N = 2
Once a day	7 (8.97)	0 (0)	6 (11.76)	-	
More than once a day	4 (5.13)	2 (11.11)	1 (1.96)	-	
Once a week	3 (3.85)	1 (5.56)	2 (3.92)	-	
Twice a week	6 (7.69)	2 (11.11)	1 (1.96)	2 (40.00)	
Three to four times a week	3 (3.85)	1 (5.56)	2 (3.92)	-	
Five to six times a week	-	-	-	-	
Once a month	6 (7.69)	1 (5.56)	4 (7.84)	1 (20.00)	
Twice a month	5 (6.41)	1 (5.56)	3 (5.88)	-	
Once every three to six months	12 (15.38)	6 (33.33)	6 (11.76)	-	
Once a year	4 (5.13)	-	3 (5.88)	1 (20.00)	
Less than once a year	26 (33.33)	4 (22.22)	20 (39.22)	2 (40.00)	
**Duration of cannabis use:**					N = 5
Less than 1 month	3 (3.85)	-	3 (5.88)	-	
1–3 months	3 (3.85)	-	3 (5.88)	-	
3–6 months	3 (3.85)	-	3 (5.88)	-	
6–9 months	2 (2.56)	-	2 (3.92)	-	
9–12 months	4 (5.13)	1 (5.56)	3 (5.88)	-	
1–2 years	14 (17.95)	5 (27.78)	7 (13.73)	2 (40.00)	
3–5 years	13 (16.67)	1 (5.56)	10 (19.61)	-	
5–10 years	10 (12.82)	4 (22.22)	6 (11.76)	-	
10–15 years	2 (2.56)	-	2 (3.92)	-	
15+ years	19 (24.36)	7 (38.89)	6 (11.76)	4 (80.00)	
Where do patients obtain their cannabis? *					N = 7
Retail cannabis store	47 (60.26)	14 (77.78)	28 (54.90)	2 (40.00)	
Online	8 (10.26)	1 (5.56)	6 (11.76)	-	
Local pharmacy (medical cannabis)	3 (3.85)	1 (5.56)	1 (1.96)	-	

**Legend**. This table summarizes the patterns of cannabis use and timing in relation to patient reported dizziness symptoms. * Some survey questions were multi-response, allowing patients to select more than one option. Patients had the choice not to answer some questions. PVD = peripheral vestibular disorder. CFVD = central/functional vestibular Disorder. NVD = non-vestibular disorder.

**Table 4:** Cannabis dosage, methods, and effects on dizziness and related symptoms.

Categories	Total N = 78 (%)	PVD N = 18 (%)	CFVD N = 51 (%)	NVD N = 5 (%)	No Response
Primary method of ingestion *					N = 6
Smoking (bong, water pipe, blunts, joints, hand pipe)	29 (37.18)	9 (50.00)	17 (33.33)	3 (60.00)	
Vaporizer	9 (11.54)	1 (5.56)	5 (9.80)	-	
Edible (not pills/tablet)	28 (35.9)	9 (50.00)	14 (27.45)	3 (60.00)	
Tablet/pills	5 (6.41)	-	5 (9.80)	-	
Cream	3 (3.85)	-	3 (5.88)	-	
Oil	22 (28.21)	6 (33.33)	13 (25.49)	1 (20.00)	
**If smoking, what is the average daily quantity used?**	**N = 29**	**N = 9**	**N = 17**	**N = 3**	N = 7
Mean, in grams	0.306	0.472	0.212	0.125	
Range, in grams	0.125				
Mode, in grams	0.125	0.125	0.125	0.125	
**Average THC content of cannabis typically used:**					N = 17
0–4%	15 (19.23)	4 (22.22)	10 (19.61)	-	
5–9%	10 (12.82)	4 (22.22)	6 (11.76)	-	
10–29%	15 (19.21)	4 (22.22)	9 (17.65)	-	
30–49%	4 (5.13)	2 (11.11)	1 (1.96)	1 (20.00)	
Greater than or equal to 50%	1 (1.28)	-	-	-	
Unsure	16 (20.51)	3 (16.67)	9 (17.65)	4 (80.00)	
**Subjective impression of cannabis use on dizziness/related symptoms:**					N = 18
Cannabis use has helped with dizziness/related symptoms	14 (18.00)	2 (11.11)	12 (23.53)	-	
Cannabis use has not helped with dizziness/related symptoms	46 (59.00)	11 (61.11)	27 (52.94)	4 (80.00)	
**To what degree do patients feel cannabis has helped dizziness-related symptoms? Scale of 1–10**	**N = 14**	**N = 2**	**N = 12**	**N = 0**	N = 0
Mode	6	-	5	-	
Median	6	6.5	6	-	
0—No help	-	-	-	-	
1—No help–little help	-	-	-	-	
2—Little help	-	-	-	-	
3—Little help–moderate help	2 (14.29)	-	2 (16.67)	-	
4—Moderate help	-	-	-	-	
5—Moderate help–large help	3 (21.43)	-	3 (25.00)	-	
6—Moderate help–large help	3 (21.43)	1 (50.00)	2 (16.67)	-	
7—Large help	3 (21.43)	1 (50.00)	2 (16.67)	-	
8—Large help–resolved dizziness	1 (7.14)	-	1 (8.33)	-	
9—Large help–resolved dizziness	1 (7.14)	-	1 (8.33)	-	
10—Resolved dizziness	1 (7.14)	-	1 (8.33)	-	
**Has cannabis worsened dizziness-related symptoms?**	**N = 46**	**N = 11**	**N = 27**	**N = 4**	N = 6
Yes	6 (13)	1 (9.09)	4 (14.81)	-	
No	34 (74)	10 (90.91)	17 (62.96)	4 (100.00)	
Which dizziness-related symptoms have cannabis-derived medications helped with? *					N = 20
Dizziness, vertigo, or balance	11 (14.1)	2 (11.11)	9 (17.65)	-	
Functional difficulties (concentration, fatigue, brain fog)	8 (10.26)	2 (11.11)	5 (9.80)	-	
Emotional difficulties (depression, anxiety, feeling upset, or fear)	17 (21.79)	5 (27.78)	11 (21.57)	-	
Headaches or migraines	9 (11.54)	-	8 (15.69)	-	
Neck pain and/or neck stiffness	14 (17.95)	1 (5.56)	12 (23.53)	-	
Sleep	28 (35.9)	11 (61.11)	16 (31.37)	1 (20.00)	
Other	10 (12.82)	-	9 (17.65)	1 (20.00)	
None	16 (20.51)	3 (16.67)	8 (15.69)	3 (60.00)	

**Legend**. This table summarizes cannabis dosages, methods of use, and effects on dizziness-related symptoms experienced by patients with dizziness. * Some survey questions were multi-response, allowing patients to select more than one option. Patients had the choice not to answer some questions. PVD = peripheral vestibular disorder. CFVD = central/functional vestibular disorder. NVD = non-vestibular disorder.

## Data Availability

The original contributions presented in this study are included in the article. Further inquiries can be directed to the corresponding author.

## Abbreviations

The following abbreviations are used in this manuscript:

AIED	Autoimmune inner ear disease
BPPV	Benign paroxysmal positional vertigo
BVH	Bilateral vestibular hypofunction
CB1	Cannabinoid receptor 1
CB2	Cannabinoid receptor 2
CBD	Cannabidiol
CBT	Cognitive behavioural therapy
CFVD	Central/functional vestibular disorder
ECS	Endocannabinoid system
NVD	Non-vestibular disorder
OHSN-REB	Ottawa Hospital Science Network Research Ethics Board
PPPD	Persistent postural–perceptual dizziness
PVD	Peripheral vestibular disorder
THC	Tetrahydrocannabinol
CAD	Canadian dollars
USD	United States dollars
